# Effects of Mind-Body Exercise in Cancer Survivors: A Systematic Review and Meta-Analysis

**DOI:** 10.1155/2020/7607161

**Published:** 2020-09-04

**Authors:** Lining Duan, Yifeng Xu, Min Li

**Affiliations:** The Medical College of Acu-Moxi and Rehabilitation, Guangzhou University of Chinese Medicine, Guangzhou 510006, China

## Abstract

**Objective:**

Mind-body exercise may have potential benefits for cancer survivors according to previous studies. We performed a systematic review and meta-analysis to summarize the published evidence and evaluate the safety and efficacy of mind-body exercise on general quality of life (QOL) and symptom management in cancer survivors.

**Methods:**

Four English language databases were systematically searched for existing randomized controlled trials (RCTs) of mind-body exercise in cancer survivors from database inception through October 23, 2019. Methodological quality was appraised with the Cochrane Risk of Bias tool. A meta-analysis of comparative effects was performed using the Review Manager v.5.3 software.

**Results:**

Fifteen studies encompassing 1461 patients were included. Analysis results showed that mind-body exercise could have a statistically significant effect on the outcomes of physical fitness, fatigue, sleep quality, depression, anxiety, and BMI, while effects on general QOL and stress were not statistically significant (all *p* > 0.05). No serious adverse events were reported.

**Conclusions:**

The current evidence demonstrates that mind-body exercise is relatively safe and modestly effective for symptom management in cancer survivors. Furthermore, randomized trials with larger sample sizes and of higher methodological quality are needed to confirm these results.

## 1. Introduction

Cancer has transformed from a fatal to a more chronic disease in many cases, and the number of survivors will reach 26 million in 2040. Over 64% of cancer patients currently could survive for at least 5 years after diagnosis [1]. Conventional treatments for cancer have shown limited efficacy and adverse consequences. Cancer survivors are more likely to suffer from secondary health problems such as fatigue, insomnia, obesity, and mental disorders [2, 3]. These problems may have negative impacts on the quality of life and physical and mental activities of cancer survivors and increasing their health care burden [4]. Therefore, it is essential to look for a safe and effective treatment to solve these problems.

Mind-body exercise, as a complementary and alternative therapy that combines body movement with mental concentration, is encouraged because it has reliable benefits in regard to improving quality of life (QOL) and relieving cancer-related symptoms [5]. Previous reviews reported that mind-body exercise interventions such as Tai Chi, yoga, and Qigong may be suitable for helping cancer patients. For example, yoga showed a trend toward a positive effect on QOL and markable improvements on anxiety, depression, and distress. Tai Chi and Qigong could alleviate subjective sleep problems, cognitive problems, and fatigue in breast cancer patients. A dance intervention was reported to have positive effects on QOL, physical activity, and vitality in cancer survivors [6–9]. However, none of these studies arrived at a definitive conclusion or evaluated the overall efficacy of all major types of mind-body exercise in a single work. In addition, more rigorous randomized controlled trials (RCTs) of mind-body exercise published in recent years were not included in previous systematic reviews. Limited available evidence could be used by caregivers who need to provide clear recommendations to cancer patients.

It is imperative that the clinical benefits of mind-body exercise are to be better understood. Therefore, we conducted a systematic review and meta-analysis to quantitatively and systematically identify the safety and efficacy of mind-body exercise in general QOL and symptom management among cancer survivors.

## 2. Materials and Methods

### 2.1. Search Strategy

This meta-analysis was conducted in accordance with the Preferred Reporting Items for Systematic Reviews and Meta-Analyses (PRISMA) guidelines [10]. Medline via PubMed, EMBASE, and the Cochrane Library databases were searched for all relevant citations published from database inception through October 23, 2019. The ClinicalTrials.gov database was searched to identify any finished but not yet published trials and any relevant trials that were still ongoing. Medical Subject Heading (MeSH) and keywords were used as the search strategy in each database. The following MeSH terms and keywords were used: “mind-body therapy” [MeSH Terms] OR “mind-body exercise” [Title/Abstract] OR “mind-body medicine” [Title/Abstract] OR “tai chi” [Title/Abstract] OR “taiji” [Title/Abstract] OR “qigong” [Title/Abstract] OR “yoga” [Title/Abstract] OR “dance” [Title/Abstract], AND “cancer” [MeSH Terms] OR “tumor” [Title/Abstract] OR “tumour” [Title/Abstract] OR “neoplasm” [Title/Abstract] OR “oncolog” [Title/Abstract], AND “random” [MeSH Terms] OR “clinical trials as topic” [MeSH Terms] OR “clinical trial” [Publication Type]. In addition, existing systematic reviews were examined to identify any additional trials.

### 2.2. Study Selection

Two of the authors (Duan and Xu) independently screened the records of the comprehensive searches by titles and abstracts, or full text as needed, to establish the eligibility of the studies. We selected studies adhering to the following criteria: (1) RCTs published in English exploring the safety and efficacy of mind-body exercise in cancer patients were assessed, regardless of publication status. (2) Participants were adults (18 years and older) with a diagnosis of any type of cancer without gender or ethnicity restrictions. (3) Studies that compared mind-body exercise with standard care or any active nonpharmaceutical control were included. All types of mind-body exercise were considered eligible including Tai Chi, Qigong, yoga, and dance. No restriction was made regarding frequency, intensity, or duration of the programme. Interventions could be supervised or home-based. However, studies with insufficient data or irrelevant outcomes were excluded. (4) The primary outcome of this meta-analysis was general QOL. The secondary outcomes were cancer-related symptoms such as physical fitness, fatigue, sleep quality, depression, anxiety, stress, and BMI. If available, therapy-related adverse events data served as a secondary outcome measure. Studies that reported only improvement rates were excluded.

### 2.3. Quality Assessment

The methodological quality of the included studies was independently appraised by 2 reviewers (Duan and Xu) according to the criteria in the Cochrane Handbook for Systematic Reviews of Interventions [11]. Each RCT was assigned a low, high, or unclear risk of bias according to sequence generation, allocation concealment, blinding of participants and outcome assessment, incomplete outcome data, selective outcome reporting, and other potential threats. If any discrepancies existed, a senior reviewer (Li) was consulted to reach an agreement. Where necessary, the authors of original studies were contacted to obtain more detailed information.

### 2.4. Data Extraction

Two reviewers (Duan and Xu) independently assessed all the studies based on the predesigned standards. Extracted data from the included studies contained the following information: general information (author name, year of publication, and research site), study characteristics (sample size, mean age, tumor type, and recent treatment), interventions (type, duration, and control details), and main outcome measures. Disagreements were rechecked by discussion with a senior reviewer (Li). Any unreported information in the study was sought by contacting the original studyʼs authors.

### 2.5. Statistical Analysis

We conducted our meta-analysis using Review Manager v.5.3 software (Cochrane Collaboration, Oxford, UK). The mean difference (MD) or standardized mean difference (SMD) with 95% confidence interval (CIs) was used to analyze continuous outcomes. We chose the SMD statistic when the outcome was assessed by the different scales. *I*^2^ statistics were calculated to assess the heterogeneity and to choose the effect model. If *I*^2^ > 50% and the *p* value of the *χ*^2^ was less than 0.1, meaning that statistical heterogeneity existed across trials, a random-effects model was used. If the pooled result included clinical heterogeneity, a subgroup analysis was used to search for the source of heterogeneity. In addition, we also conducted a sensitivity analysis to evaluate the stability of the pooled results.

## 3. Results

### 3.1. Study Identification and Selection

**Figure 1:**
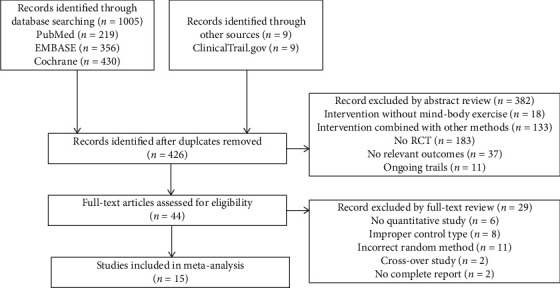
Flowchart of the results of the literature research.

We retrieved a total of 1014 citations through database searches. After removing duplicate entries, we screened 426 articles and excluded 382 articles based on the abstract and title. We then assessed the full-text versions of the remaining articles and found 15 studies eligible for inclusion [12–26] (Figure 1).

### 3.2. Characteristics of the Included Studies

A total of 15 published RCTs with 1461 participants that met our inclusion criteria were included in this analysis (Table 1). The mean age of the included participants ranged from 44 to 66 years with the intervention duration in the range of 3–24 weeks, and the sample sizes varied from 16 to 410. The major types of mind-body exercise interventions included Tai Chi, Qigong, yoga, and dance. The exercise duration generally ranged from 20 to 90 minutes per session with a frequency of 2 times a week to once a day. The exercise intensity depended on the exercise component and the endurance capacity of the patients. Three studies used active controls including health education, daily physical activity, and low-impact exercise. The other studies used usual care as the control group.

### 3.3. Quality Assessment

**Figure 2:**
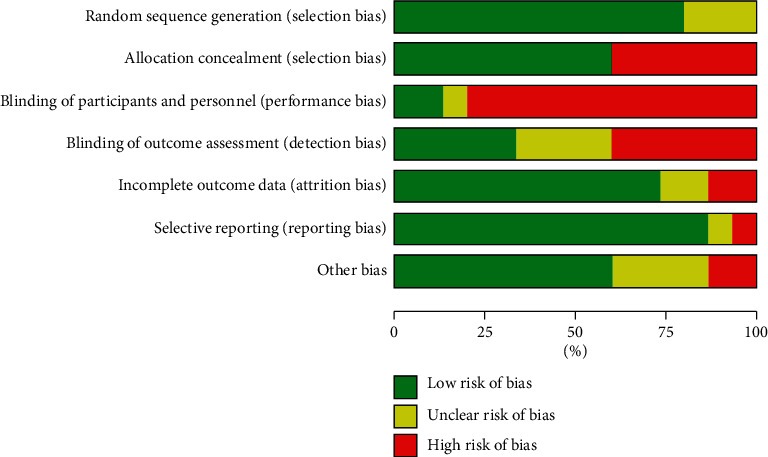
Risk of bias.

**Figure 3:**
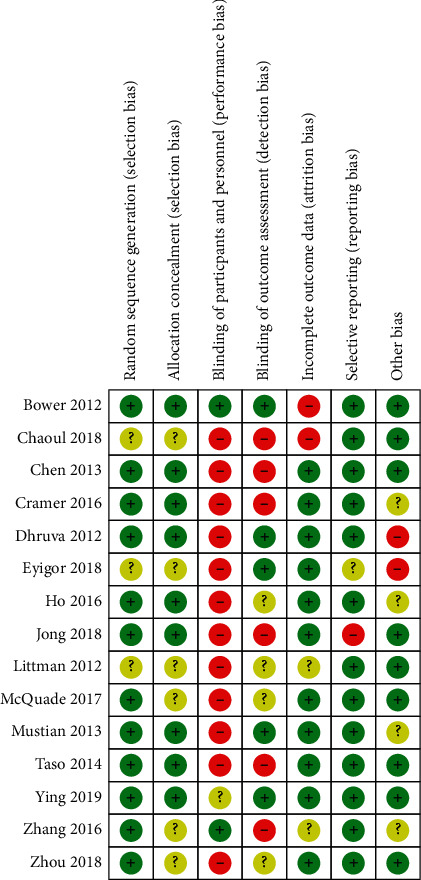
Risk of bias summary.

Among the 15 RCTs included, 12 studies (80%) [12, 14–16, 18, 19, 21–26] reported adequate random sequence generation, and 9 studies (60%) [12, 14–16, 18, 19, 22–24] reported the use of allocation concealment methods. Only one study [12] reported blinding of participants and outcome assessment, probably because mind-body exercise could be difficult to implement the methods of blinding since it is a nonpharmaceutical therapy. A total of 12 studies (80%) [14–19, 21–24, 26] had complete outcome data. One study [19] was judged to have a high risk of selective reporting biases, and two studies [16, 17] had a high risk of other bias. Overall, 11 studies (73.3%) [12, 14–16, 18, 19, 21–24, 26] were recognized as having a low risk of methodological quality (Figures 2 and 3).

### 3.4. Analysis of Outcomes

#### 3.4.1. Primary Outcomes

**Figure 4:**
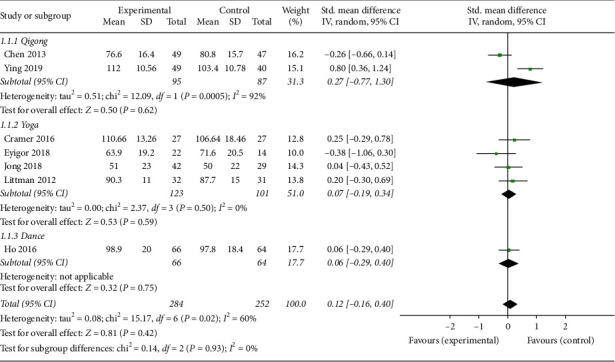
Forest plot of the mind-body exercise group versus the control group: general QOL.

*(1) General QOL*. Seven studies [14, 15, 17–20, 24] reported general QOL outcomes. Functional Assessment of Cancer Therapy-General (FACT-G), Functional Assessment of Cancer Therapy-Breast (FACT-B), and European Organization for Research and Treatment of Cancer Quality of Life Questionnaire (EORTC-QLQ-C-30) were each used in two studies to measure general QOL. Functional Assessment of Cancer Therapy-Colorectal (FACT-C) was used in only one study. The overall pooled results revealed that mind-body exercise had no significant improvement of general QOL compared with the control group (SMD: 0.12; 95% CI: 0.16–0.40; *p*=0.42). Heterogeneity existed among the trials (*p*=0.02; *I*^2^ = 60%). No significant differences were found among the subgroups (*p*=0.93; *I*^2^ = 0%). No subgroup showed a statistically significant effect on general QOL when compared with the control group (*p*=0.62, *p*=0.59, and *p*=0.75, respectively) (Figure 4).

#### 3.4.2. Secondary Outcomes

**Figure 5:**
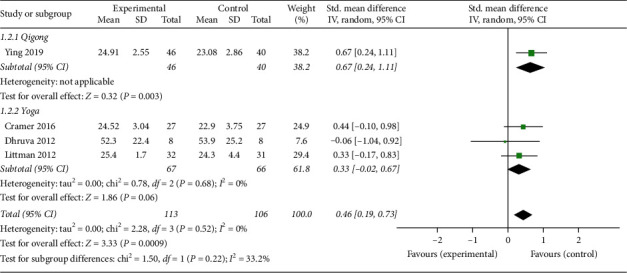
Forest plot of the mind-body exercise group versus the control group: physical fitness.

*(1) Physical Fitness*. Four studies [15, 16, 20, 24] reported physical fitness outcomes. Physical fitness was evaluated by clinical assessment scales including physical well-being of Functional Assessment of Cancer Therapy-Breast (FACT-B), physical well-being of Functional Assessment of Cancer Therapy-Colorectal (FACT-C), 12-Item Short-Form Health Survey (SF-12), and physical well-being of Functional Assessment of Cancer Therapy-General (FACT-G). The overall pooled results revealed significant improvements of physical fitness in response to mind-body exercise (SMD: 0.46; 95% CI: 0.19–0.73; *p* < 0.01). Heterogeneity did not exist among the trials (*p*=0.52; *I*^2^ = 0%). No significant difference was found among the subgroups (*p*=0.22; *I*^2^ = 33.2%). The Qigong group was significantly different from the control group in terms of physical fitness (SMD: 0.67; 95% CI: 0.24–1.11; *p*=0.003) (Figure 5).

**Figure 6:**
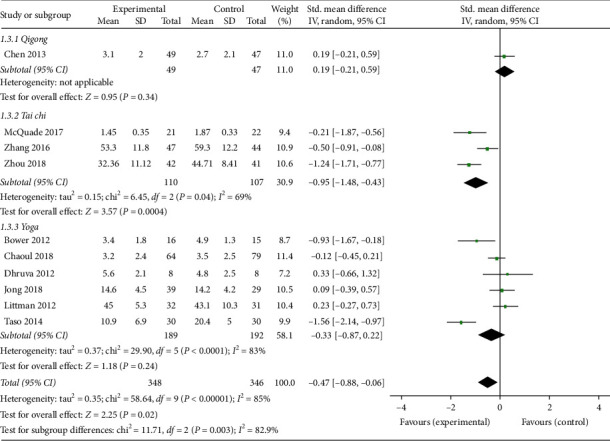
Forest plot of the mind-body exercise group versus the control group: fatigue.

*(2) Fatigue*. Ten studies [12–14, 16, 19–21, 23, 25, 26] reported fatigue outcomes, and four of those used Brief Fatigue Inventory (BFI), two used Multidimensional Fatigue Symptom Inventory-Short Form (MFSI-SF), one used Fatigue Symptom Inventory (FSI), two used Functional Assessment of Chronic Illness Therapy-Fatigue (FACIT-F), one used revised Piper Fatigue Scale (PFS), and one used Multidimensional Fatigue Inventory (MFI) to evaluated fatigue. The overall pooled results revealed that mind-body exercise resulted in a significant relief of fatigue (SMD: −0.47; 95% CI: −0.88 to −0.06; *p*=0.22). Heterogeneity existed among the trials (*p* < 0.01; *I*^2^ = 85%). Significant difference was found among the subgroups (*p*=0.003; *I*^2^ = 82.9%). Only the Tai Chi group was significantly different from the control group in terms of fatigue (SMD: −0.95; 95% CI: −1.48 to −0.43; *p* < 0.01) (Figure 6).

**Figure 7:**
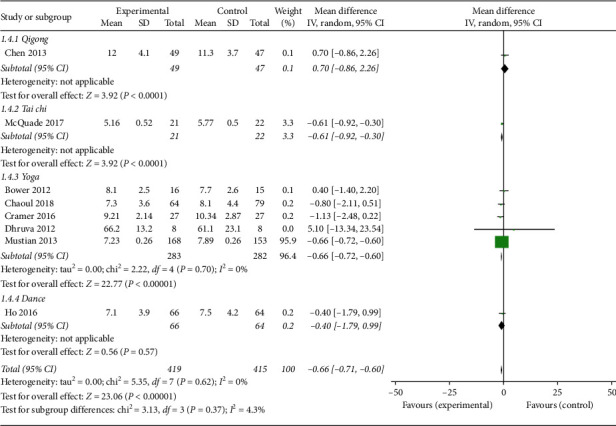
Forest plot of the mind-body exercise group versus the control group: sleep quality.

*(3) Sleep Quality*. Eight studies [12–16, 18, 21, 22] reported sleep quality outcomes, and seven of those used Pittsburgh Sleep Quality Index (PSQI) and one used the General Sleep Disturbance Scale (GSDS) to measure sleep quality. The overall pooled results revealed statistically significant improvements of sleep quality in response to mind-body exercise (MD: −0.66; 95% CI: −0.71 to −0.60; *p* < 0.01). Heterogeneity did not exist among the trials (*p*=0.62; *I*^2^ = 0%). Similarly, no significant differences were found among the subgroups (*p*=0.37; *I*^2^ = 4.3%). The Tai Chi and yoga subgroups showed a significant effect on sleep quality (MD: −0.61; 95% CI = −0.92 to −0.30; *p* < 0.01, and MD: −0.66; 95% CI: −0.72 to −0.60; *p* < 0.01, respectively) (Figure 7).

**Figure 8:**
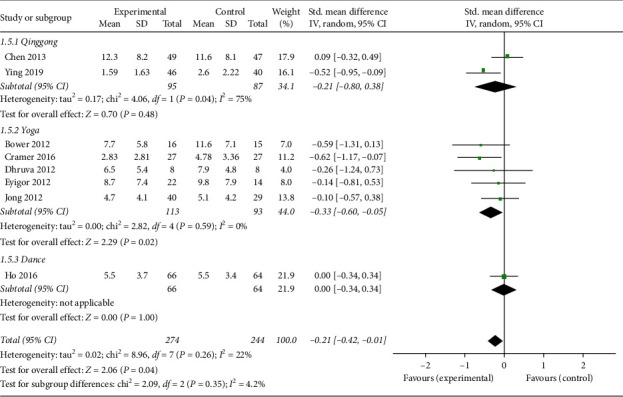
Forest plot of the mind-body exercise group versus the control group: depression.

*(4) Depression*. Eight studies [12, 14–19, 24] reported depression outcomes, and four of those used Hospital Anxiety and Depression Scale (HADS), two used Beck Depression Inventory (BDI), one used Center for Epidemiologic Studies Depression (CESD), and one used Patient Health Questionnaire-9 (PHQ-9) to evaluate depression. The overall pooled results revealed that mind-body exercise led to significant relief from depression (SMD: −0.21; 95% CI = −0.42 to −0.01; *p*=0.04). Heterogeneity did not exist for the trials (*p*=0.26, *I*^2^ = 22%). No significant differences were found among the subgroups (*p*=0.35, *I*^2^ = 4.2%). However, only the yoga group was significantly different from the control group in terms of depression (SMD: −0.33; 95% CI: −0.60 to −0.05; *p*=0.02) (Figure 8).

**Figure 9:**
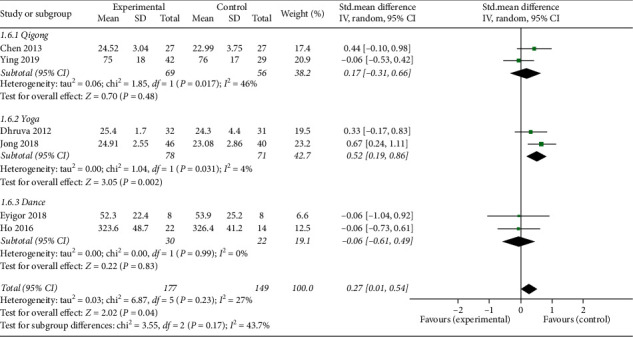
Forest plot of the mind-body exercise group versus the control group: anxiety.

*(5) Anxiety*. Six studies [14, 16–19, 24] reported anxiety outcomes, which were evaluated by different assessment scales including the Center for Epidemiologic Studies Depression Scale (CESD), Hospital Anxiety and Depression Scale (HADS), and Generalized Anxiety Disorder-7 scale (GAD-7). The overall pooled results revealed that mind-body exercise had a statistically significant effect in relieving anxiety (SMD: 0.27; 95% CI: 0.01–0.54; *p*=0.04). Heterogeneity did not exist among the trials (*p*=0.23, *I*^2^ = 27%). No significant differences were found among the subgroups (*p*=0.17, *I*^2^ = 43.7%). However, only the yoga group was significantly different from the control group in terms of anxiety (SMD: 0.52; 95% CI: 0.19–0.86; *p*=0.002) (Figure 9).

**Figure 10:**
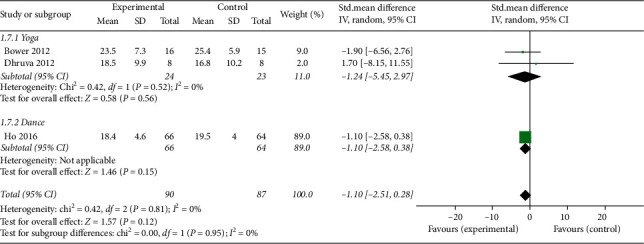
Forest plot of the mind-body exercise group versus the control group: stress.

*(6) Stress*. Three studies [12, 16, 18] reported stress outcomes evaluated by the Perceived Stress Scale (PSS). The overall effect of mind-body exercise on stress revealed no significant difference compared with the control group (MD: −1.12; 95% CI: −2.51 to 0.28; *p*=0.12). Heterogeneity did not exist among the trials (*p*=0.81; *I*^2^ = 0%). No significant differences were found among the subgroups (*p*=0.95; *I*^2^ = 0%) (Figure 10).

**Figure 11:**
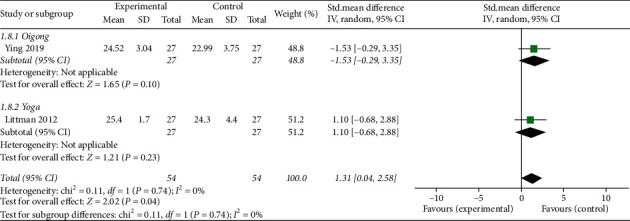
Forest plot of mind-body exercise group versus control group: BMI.

*(7) BMI*. Two studies [20, 24] reported body mass index (BMI) outcomes. The overall pooled results showed a statistically significant advantage for the mind-body exercise group in regard to BMI (MD: 1.31; 95% CI: 0.04 to 2.58; *p*=0.04). Heterogeneity did not exist among the trials (*p*=0.74; *I*^2^ = 0%). No significant differences were found among the subgroups (*p*=0.74; *I*^2^ = 0%) (Figure 11).

#### 3.4.3. Adverse Events

Four studies [12, 15, 19, 22] reported adverse events. Only one study reported that one participant experienced a transient back spasm during the exercise class, and she was able to return to class and completed the intervention after evaluation by her physician. All of the others studies reported no adverse events related to the treatment. In all of the trials included, no dropouts were attributed to adverse effects associated with mind-body exercise interventions.

### 3.5. Sensitivity Analysis

We performed sensitivity analyses based on excluding studies with low quality, small sample sizes, and the trial with active controls. First, we excluded two studies [17, 20] with low quality. The pooled results showed that there was no difference between the intervention group and the control group (SMD: 0.17; 95% CI: −0.18–0.52; *p*=0.34). Second, we excluded two studies [15, 17] with small sample sizes. There was no difference in the results after their exclusion (SMD: 0.16; 95% CI: −0.18 to 0.50; *p*=0.36). Finally, we eliminated the study [24] with daily physical activity as the control intervention. After this step, the statistical heterogeneity disappeared, but there was no difference in the results after their exclusion (SMD: −0.00; 95% CI: −0.19 to 0.18; *p*=0.97).

**Figure 12:**
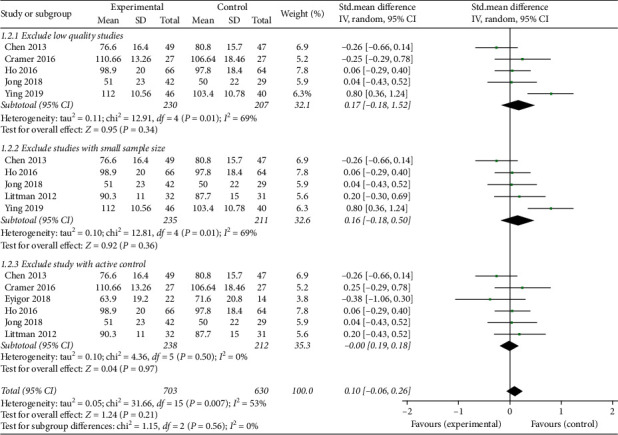
Forest plot of the sensitivity analysis of the primary outcome.

A total of three sensitivity analyses reached similar results as the overall analysis for the primary outcome, which reflected that our results are stable and reliable (Figure 12).

### 3.6. Publication Bias

**Figure 13:**
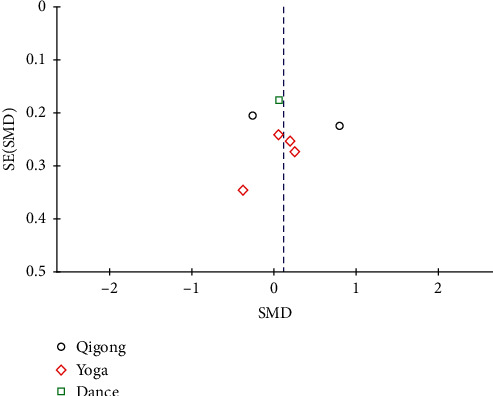
Funnel plot of publication bias of the primary outcome.

No significant publication bias was found from the funnel plot (Figure 13).

## 4. Discussion

### 4.1. Summary of Findings

We performed a systematic review and meta-analysis that included 15 published RCTs with 1461 individuals. The articles reported comparisons of mind-body exercise against either standard care or an active control in cancer survivors. We evaluated the safety and efficacy of mind-body exercise on general QOL and symptom management including physical fitness, fatigue, sleep quality, depression, anxiety, stress, and BMI. Our study produced several important findings.

Our meta-analysis demonstrated significant effects of mind-body exercise in cancer survivors on physical fitness, fatigue, sleep quality, depression, anxiety, and BMI. However, no evidence was found for mind-body exercise improving general QOL or stress levels. These results were based on the clinical manifestations as measured by standard assessment instruments. The assessment instruments used in these studies were of high consistency, reliability, and construct validity, revealing that they are reliable measures of cancer-related symptoms. Consistent with our study, recent studies also reported that mind-body exercise could improve sleep outcomes in cancer patients with poor sleep quality after intervention [27, 28].

Subgroup analyses of the different types of mind-body exercise suggested that Qigong might effectively improve physical fitness, Tai Chi is beneficial for alleviating fatigue and sleep problems, and yoga could result in meaningful benefits for mental disorders in cancer patients. These results are consistent with previous reviews exploring individual types of mind-body exercise interventions for their effects on symptom management in cancer patients [29–32]. Our results also showed that subgroup differences among the various types of interventions were significant, which means that the type of mind-body exercise intervention might have a measurable impact on its efficacy for cancer survivors. Tai Chi, Qigong, and yoga all belong to mind-body exercise, which combined some forms of movements that focus on breathing with a calm state of mind [33]. However, Tai Chi and Qigong have their origins as martial arts based on traditional Chinese medicine, which rooted in the ancient philosophy of naive materialism and natural dialectics [34]. Yoga is a physical, mental, and spiritual discipline originating from ancient India, which could deliver practitioners from suffering or disease [35]. Different types of mind-body exercises are different in training methods and essentials, postures, movement characteristics, purpose, and function [36]. Therefore, different types of mind-body exercise interventions might target different symptoms of cancer. In addition, the outcomes of our subgroup analyses had similar conclusions to the overall analysis, which reflects that our evidence is stable and reliable.

The adverse events data demonstrated that mind-body exercise was a relatively safe therapy for individuals with cancer that is not associated with serious adverse events. However, caution should be applied because of certain limitations. Only four studies reported adverse events, and we suggest that future RCTs should report more information about adverse events.

Both physical fitness and mental function have been shown to be impaired in individuals with cancer [37, 38]. Mind-body exercise, as a complementary and alternative therapy that combines coordinated physical movements and regulated attention and consciousness, has measurable effectiveness in treating diseases and secondary conditions, including mental disorders and physical problems [39]. Patients who used mind-body exercise interventions may experience more positive affective states, more social group support, and fewer physical symptoms. These factors may singly or together provide benefit to cancer survivors [29].

Mind-body exercise may relieve the cancer-related symptoms via the following mechanisms. First, mind-body exercise plays a positive role in the network involved in the regulation of attention, emotion, and executive function. Recent studies suggested that mind-body exercises were able to target different brain systems that are involved in the regulation of attention, emotion, mood, and executive cognition [40]. Positive effects of mind-body exercises were found in brain regions involved in body awareness, attention, and the integration of emotion and sensory processing [41]. Second, mind-body exercise could increase the inhibition of sympathetic responses to stress, resulting in regulation of the expression of affective, autonomic, hormonal, and immune responses through neurovisceral feedback via the vagus nerve to the prefrontal cortex and limbic system [42]. Mind-body practice may exert further beneficial effects by enhancing cell-mediated and mucosal immunity [43]. Third, the proven efficacy of mind-body exercise in anti-inflammation and antioxidant effects could provide benefits for cancer survivors. Recent studies pointed out that mind-body exercise could provoke the moderate elevations of anti-inflammatory factor interleukin-10 and inhibit the expression of proinflammatory factors including tumor necrosis factor-*α* and interleukin-1*β* [44]. Furthermore, it was reported that mind-body exercise could decrease the level of malondialdehyde and increase the levels of catalase, superoxide dismutase, catalase, and glutathione peroxidase in serum of prostate cancer patients [45]. In addition, a clinical research showed that greater frequency of mind-body exercise was related to higher posttreatment insulin-like growth factor 1, a biomarker associated with human longevity [46]. Moreover, it was reported that mind-body exercise could counteract hippocampal vulnerability to neurotoxicity, which could prevent cognitive impairment and improve QOL [47]. Consistent with previous studies, our findings indicated that there were clinically measurable effects on alleviating cancer-related symptoms in favor of mind-body exercise.

To our knowledge, our review is the first study that included all types of major mind-body exercise and comprehensively evaluated their safety and overall efficacy in cancer survivors. Previous reviews mostly assessed only one type of mind-body exercise or involved mind-body exercise as a component, which means that it may be difficult to examine the overall effects comprehensively and accurately. Our study focused on the overall effects of mind-body exercise on general QOL and symptom management in cancer patients, which was different from other meta-analyses in this field.

Regarding the quality of the included studies, 73.3% was evaluated as high-quality studies, which indicates that the quality of evidence in our study is moderate. However, bias existed in the domains of performance and detection bias, which might weaken the power and credibility of the results.

### 4.2. Implications for Clinical Practise

From a clinical perspective, there are some potential clinical implications of our findings. First, the patients with different types and in different stages of cancer could benefit from mind-body exercise since it could improve physical fitness, decrease BMI, and alleviate fatigue, sleep problems, depression, and anxiety as an evidence-based complementary and alternative therapy. Second, among the major mind-body exercise types, Qigong could improve physical fitness effectively, Tai Chi provided benefits for sleep problems and fatigue, and yoga might be most suitable for alleviating mental disorders. Caregivers could provide evidence-based recommendations due to different symptoms troubling specific patients. Finally, the exercise duration and intensity should consider the endurance capacity of patients. Thus, given its efficacy, safety, and relatively low cost, implementation of mind-body exercise could be promoted as an effective therapy for cancer survivors.

### 4.3. Limitations

Several limitations of this meta-analysis need to be taken into consideration. First, the relatively small number of eligible RCTs and their typically small sample sizes in the analysis of the primary outcome may lead to negative results and limited the precision of the findings. Second, we could not conduct subgroup analyses according to gender, age, or region due to the shortage of original studies. The number of studies included for each type of mind-body exercise was small, and thus, it seemed difficult to properly assess publication bias. Third, although blinding of participants or care providers may be difficult in mind-body exercise interventions, the high risk of performance and detection bias might weaken the strength of the evidence. Additionally, although the trials included in this meta-analysis covered various cancer types, the majority of the evidence is based on patients with breast cancer. Finally, we only included studies published in English, which may influence our results to some extent and limited the generalizability of our findings.

## 5. Conclusion

In conclusion, the findings of this systematic review and meta-analysis suggests that mind-body exercise is a relatively safe and modestly effective therapy that could lead to measurable improvements in symptom management in cancer survivors, particularly in physical fitness, fatigue, sleep quality, depression, anxiety, and BMI, but it had no statistically advantage for general QOL and stress. Further randomized trials with larger sample sizes and of a higher methodological quality, especially those with a carefully blinded design, are needed for confirmation of these findings in the future.

## Figures and Tables

**Table 1:** Characteristics of included studies.

Study	Cancer type	Mean age (E/C)	Sample size	Experiment	Control	Duration	Outcome measures
Bower, USA	Breast	54.4 ± 5.7/53.3 ± 4.9	31	Yoga	Health education	12 w	FSI, PSQI, BDI-II, and PSS
Chaoul, USA	Breast	49.5 ± 9.8/49 ± 10.1	159	Yoga	Usual care	12 w	BFI and PSQI
Chen, China	Breast	45.3 ± 6.3/44.7 ± 9.7	96	Qigong	Usual care	5 w	FACT-G, BFI, PSQI, and CESD
Cramer, Germany	Colorectal	68.7 ± 9.13/67.81 ± 10.3	54	Yoga	Usual care	10 w	FACT-C, PSQI, and HADS
Dhruva, USA	Mixed	52.4 ± 14.6/56.0 ± 11.9	16	Yoga	Usual care	4 w	SF-12, PFS, GSDS, HADS, and PSS
Eyigor, Turkey	Breast	52.3 ± 9.5/51.5 ± 7.3	42	Yoga	Usual care	10 w	EORTC-QLQ-C-30, and BDI
Ho, China	Breast	48.6 ± 7.7/49.1 ± 8.7	139	Dance	Usual care	3 w	FACT-B, PSQI, HADS, HADS, and PSS
Jong, Netherlands	Breast	51 ± 8.0/51 ± 7.3	83	Yoga	Standard care	12 w	EORTC-QLQ-C-30, MFI, and HADS
Littman, USA	Breast	60.6 ± 7.1/58.2 ± 8.8	63	Yoga	Usual care	24 w	FACT-G, FACIT-F, and BMI
McQua, USA	Prostate	62.2 ± 7.4/66.0 ± 8.4	43	Tai Chi	Usual care	8 w	BFI and PSQI
Mustian, USA	Mixed	54.3 ± 0.77/54.0 ± 0.67	410	Yoga	Standard care	4 w	PSQI
Taso, China	Breast	49.27 ± 10.23	60	Yoga	Standard care	8 w	BFI
Ying, China	Breast	54.09 ± 7.76	86	Qigong	Daily physical activity	24 w	FACT-B, PHQ-9, GAD-7, and BMI
Zhang, China	Lung	62.8	96	Tai Chi	Low-impact exercise	12 w	MFSI-SF
Zhou, China	Nasopharyngeal carcinoma	NR	83	Tai Chi	Usual care	4 w	MFSI-SF

E, experiment; C, control; BDI, Beck Depression Inventory; BFI, Brief Fatigue Inventory; BMI, body mass index; CESD, Center for Epidemiologic Studies Depression; EORTC-QLQ-C-30, European Organization for Research and Treatment of Cancer Quality of Life Questionnaire; FACT-B, Functional Assessment of Cancer Therapy-Breast; FACT-C, Functional Assessment of Cancer Therapy-Colorectal; FACT-G, Functional Assessment of Cancer Therapy-General; FACIT-F, Functional Assessment of Chronic Illness Therapy-Fatigue; FSI, Fatigue Symptom Inventory; GAD-7, Generalized Anxiety Disorder-7 scale; GSDS, General Sleep Disturbance Scale; HADS, Hospital Anxiety and Depression Scale; MFI, Multidimensional Fatigue Inventory; MFSI-SF, Multidimensional Fatigue Symptom Inventory-Short Form; PHQ-9, Patient Health Questionnaire-9; PSQI, Pittsburgh Sleep Quality Index; PFS, revised Piper Fatigue Scale; PSS, Perceived Stress Scale; SF-12, 12-Item Short-Form Health Survey.

## Data Availability

The data used to support the findings of this study are available from the corresponding author upon request.
